# Nr4a2, A Key Factor Controlling the Development and Functional Maintenance of Forebrain *Car3* Neurons

**DOI:** 10.1007/s12264-025-01496-z

**Published:** 2025-09-08

**Authors:** Yun-Chao Tao, Li Zhao, Qiong Zhang, Xi-Yue Liu, Wei-Tang Liu, Ze-Xuan Li, Ling Hu, Lei Zhang, Jia-Yin Chen, Yu-Qiang Ding, Ning-Ning Song

**Affiliations:** 1 State Key Laboratory of Brain Function and Disorders, MOE Frontiers Center for Brain Science, Institutes of Brain Science, Fudan University, Shanghai, 200032 China; 2 Laboratory Animal Center, Fudan University, Shanghai, 200032 China; 3 Shanghai Institute of Infectious Diseases and Biosecurity, Fudan University, Shanghai, 200032 China; 4 Shanghai Yangzhi Rehabilitation Hospital (Shanghai Sunshine Rehabilitation Center), Tongji University School of Medicine, Shanghai, 201619 China; 5 Clinical Center for Brain and Spinal Cord Research, Tongji University, Shanghai, 200092 China; 6 Huashan Institute of Medicine (HS-IOM), Huashan Hospital, Fudan University, Shanghai, 200040 China

**Keywords:** *Nr4a2*, *Car3*, Development, Function maintenance, Anxiety

## Abstract

*Nr4a2* (*Nurr1*) is well known to be vital for midbrain dopaminergic neurons. Recent single-cell RNA analyses reveal that *Nr4a2* is expressed in lateral cerebral regions, within neurons named L4/L5/L6 IT *Car3*. These neurons have attracted intense attention for the molecular mechanisms underlying their development and functions. We classified *Car3* neurons into neocortical (Ncx-*Car3*), claustral (CLA-*Car3*), and dorsal endopiriform nucleus (dEn-*Car3*) subpopulations, focusing on the characterization of Ncx-*Car3* neurons. These neurons exhibit distinct birthdates and migratory morphologies compared to CLA- and dEn-*Car3* neurons, but share a common transcriptomic profile when *Nr4a2* is deleted at the embryonic stage or in adulthood. Notably, *Nr4a2* misexpression ectopically induces *Car3*-enriched genes *in vivo*. Mice lacking *Nr4a2* in *Car3* ensembles during the embryonic stage or in adulthood display hyperactivity and reduced anxiety-like behaviors. Therefore, our results demonstrate that Nr4a2 is a key factor in regulating the development and functional maintenance of the forebrain *Car3* neurons.

**Supplementary Information:**

The online version contains supplementary material available at 10.1007/s12264-025-01496-z.

## Introduction

Nuclear receptor subfamily 4 group A member 2 (Nr4a2, also known as Nurr1) is a well-known transcription factor for its critical roles in the transcriptional control of the differentiation and survival of midbrain dopaminergic neurons [1–4], and thus is believed to be an important treatment target of Parkinson’s disease [5–9]. *Nr4a2* has intense expression in the forebrain, and many Nr4a2-positive neurons are located in the deep layers of the lateral neocortex. While it is thought to have a protective effect against lipopolysaccharide (LPS)-induced behavior variation in the anterior cingulate cortex [10], the role of Nr4a2 in other forebrain regions remains elusive.

In recent years, several single-cell RNA sequencing analyses have identified a special subclass of cortical excitatory neurons, named layer 4/5/6 intratelencephalic *Car3* (L4/5/6 IT *Car3*) neurons, located in deep neocortical layers [11–13]. Since this group of neurons has a transcriptome profile highly similar to the neurons in the claustrum (CLA) and dorsal endopiriform nucleus (dEn), they were called *Car3* neurons as a whole [12]. In order to distinguish accurately between them, we named the *Car3* neurons as three subpopulations: Ncx-*Car3*, CLA-*Car3*, and dEn-*Car3* neurons, based on their anatomical locations. The Ncx-*Car3* neurons are highly conserved in evolution [14], implying their necessity in regulating brain function. However, the developmental mechanism as well as the function of Ncx-*Car3* neurons has yet to be explored.

Here, we report the special distribution pattern and developmental characteristics of Ncx-*Car3* neurons and knocked out the *Nr4a2* gene at the embryonic stage or in the postnatal period to differentiate its roles in the development and regulation of brain functions in adulthood. This showed that *Nr4a2* is required for the expression of *Car3* neuron-enriched genes during embryonic development and in the mature brain. In particular, *Nr4a2* misexpression can induce ectopic expression of those *Car3*-enriched genes in the neocortex of wild-type mice. Our behavioral examination revealed the correlation between *Nr4a2* deficiency in the *Car3* ensemble with low anxiety-like behaviors and hyperactivity. Together, we identified Nr4a2 as a key regulator of the Ncx-*Car3* neurons in the aspects of neural development, as well as hyperactivity and anxiety-related states.

## Materials and Methods

### Animals

*Nr4a2*^flox/flox^ mice were generated as previously described [15]. *Nr4a2*^CreER^ mice (*Nr4a2*-P2A-Cre^ERT2^-IRES-EGFP mice) and *Gnb4*^CreER^ mice (*Gnb4*-P2A-Cre^ERT2^-P2A-mEGFP mice) were constructed through CRISPR/Cas9 techniques based on a C57BL/6 background. For labeling Cre-expressing cells, *Nr4a2*^CreER^ mice or *Gnb4*^CreER^ mice were crossed with Ai14 (B6.Cg-Gt(ROSA)26Sor^tm14(CAG-tdTomato)Hze^/J) or Ai3 (B6.Cg-Gt(ROSA)26Sor^tm3(CAG-EYFP)Hze^/J) reporter mice to obtain *Nr4a2*^CreER^;Ai14 or *Gnb4*^CreER^;Ai3 mice. To conditionally knock out the *Nr4a2* gene in the forebrain or restrictively in the *Car3* population, *Nr4a2*^flox/flox^ mice were crossed with *Emx1*^Cre^ mice [16] or *Gnb4*^CreER^ mice, and *Nr4a2*^*Emx1*^ cKO (*Emx1*^Cre^; *Nr4a2*^flox/flox^) or *Nr4a2*^*Gnb4*^ icKO (*Gnb4*^CreER^;*Nr4a2*^flox/flox^) mice were obtained. Other genotypes (i.e., *Nr4a2*^flox/flox^ and *Nr4a2*^flox/+^) from the same litter were used as control mice. All the animals were maintained in a specific pathogen-free facility and provided regular rodent chow and water *ad libitum* under a 12 h light/dark cycle (lights on at 07:00). The day of vaginal plug detection was assigned as embryonic day 0.5 (E0.5), and the day of birth was recorded as postnatal day 0 (P0). No notable differences based on sex were observed, and data were pooled between sexes if not specifically mentioned. Animal care practices and all experiments were reviewed and approved by the Laboratory Animal Committee of Fudan University, China.

### Tamoxifen Administration

Tamoxifen (T5648) was purchased from Sigma-Aldrich. To induce Cre activity, mice were administered one dose of tamoxifen (100 mg/kg of body weight) intragastrically. To knock out *Nr4a2* in forebrain *Car3* neurons in adulthood, mice were administered five doses of tamoxifen intragastrically (200 mg/kg of body weight) at an interval of two days, as shown in Fig. 4C.

### Immunohistochemistry, BrdU Labeling, *In situ* Hybridization, and Nissl Staining

Anaesthetized mice were perfused with 0.01 mol/L phosphate-buffered saline (PBS) followed by 4% PFA in PBS, and then the brains were dissected out and postfixed overnight. After cryoprotection in 20% sucrose solutions, brain sections were cut at 20-30 μm on a cryostat (RWD, Shenzhen, China).

Immunohistochemistry and BrdU labeling were applied as previously described [15]. The following primary antibodies were used: mouse anti-Nr4a2 (1:300; Cat.# ab41917, Abcam), rat anti-Ctip2 (1:300; Cat.# ab18465, Abcam), guinea pig anti-Tle4 (1:200; Cat.# OB-PGP086, Oasis), rabbit anti-mCherry/tdTomato (1:800; Cat.# OB-PRB013, Oasis), goat anti-GFP (1:1000; Cat.# NBP100-1770, Novus), rat anti-BrdU (1:2000; Cat.# OBT0030G, Accurate Chemical & Scientific Corp.), and rabbit anti-cleaved caspase3 (1:500; Cat.# 9664, Cell Signaling Technology). For Nissl staining, the sections were stained with Cresyl Violet.

*In situ* hybridization (ISH) used digoxigenin UTP-labeled probes, as previously described [17]. *Rorβ*, *Ctip2*, *Pcp4*, *Nr4a2*, *Gnb4*, *Gfra1*, *Ntng2*, *Lxn*, *Sstr2*, *Lmo4*, *Lhx2*, *PlxnD1,* and *Foxp2* RNA probes were constructed according to the Allen Brain Atlas.

For the combination of ISH and immunostaining, sections were hybridized with RNA probes first, as reported in our previous study [17]. After visualization for mRNA, sections were incubated with mouse anti-Nr4a2, guinea pig anti-Tle4, goat anti-GFP, or rabbit anti-mCherry/tdTomato antibodies overnight, respectively. Then the sections were incubated with specific biotinylated secondary antibodies at room temperature for 2 h. After that, the sections were processed using the ABC kit (1:200; Vector Laboratories) for 1 h and were incubated with diaminobenzidine and H_2_O_2_ (3%) to visualize immunoreactivity.

### Dual-Luciferase Reporter Assay

The pGL4-promoter plasmids were homemade. The fragments of target gene promoters, which contained Nr4a2-binding sites, were cloned into the multiple cloning sites of the pGL4.10 plasmids, respectively. A total of 5 × 10^8^ HEK293T cells were seeded in each 48-well plate and then transfected using Lipo8000 transfection reagent (Beyotime Biotechnology, Shanghai, China). A mixture of 120 ng pGL4-promoter plasmids (or pGL4.10-empty plasmids for negative control) and 5 ng *Renilla* luciferase-expressing plasmids, with or without 120 ng *Nr4a2* overexpression plasmids, was co-transfected into the cells. About 24 h after transfection, the cells were harvested. Firefly and *Renilla* luciferase activities were measured using a dual luciferase Reporter Assay System (E1910, Promega). The ratio of firefly luciferase readouts and *Renilla* luciferase readouts was calculated to represent the activity of the reporter vector. Three replicate wells were made for each transfection. Each experiment was repeated at least three times.

The primer sequences for the fragments of the target gene promoters were as follows:

- Gnb4-F: TGCACAGGATTATGAGGGAGG
- Gnb4-R: CCACTTTGACAGACGGTTGC
- Gfra1-F: CAGATATGGTAACCATGGTCAT
- Gfra1-R: TGCACAAGGGCTCTTTCTT
- Ntng2-F: GCCCATCTCTAAACATGTAACTCA
- Ntng2-R: TGCACACAACCGCATACCAG

### *In utero* Electroporation

For misexpressing *Nr4a2* in the cerebral cortex, pCAG-EGFP plasmids (1 μg/μL) with or without pCAG-Flag-*Nr4a2* plasmids (2 μg/μL) were injected directly into the lateral ventricles of wild-type embryos at E14.5. Five square electrical pulses (36 V) with 50-ms duration were then delivered through the uterus at 1-s intervals using forceps-type electrodes, connected with an electroporator (ECM830, BTX, Holliston, MA, USA). Pups were collected at P7 for analysis. At least three animals for each group were used.

### Behavioral Tests

Adult (>2 months old) male mice were used in the following behavioral tests. All behavioral experiments were performed during the light phase in a soundproof room with a neutral environment. All mice were given a 30-min habituation period before each behavioral test. There was at least 1 day for the animals to rest between different tests. The experimenter was blind to the group identity of the tested mice. All the recorded movies were analyzed through EthoVision XT 17 software (Noldus, Wageningen, the Netherlands) unless otherwise specified.

#### Open Field Test

The open field test was carried out within a computer-operated detecting and analysis apparatus (Fusion software & SuperFlex Open Field system, Omnitech Electronics, Columbus, OH, USA). Each mouse was placed in a square field (40 cm × 40 cm) with an enclosed wall (30 cm) made of transparent plexiglass. The experiment lasted 30 min for *Nr4a2*^*Emx1*^ cKO mice or 60 min for *Nr4a2*^*Gnb4*^ icKO mice. The total distance traveled, ambulatory time, and average velocity during ambulation were recorded.

#### Elevated Plus Maze Test

The elevated plus maze consisted of two open arms (30 cm × 5 cm), two enclosed arms (30 cm × 5 cm × 15 cm), and a central platform (5 cm × 5 cm), which was elevated 40 cm above the ground. Each mouse was placed on the central platform facing one of the open arms to start and observed for 5 min. The time spent in each arm and the frequency of entries into each arm were recorded. An entry was defined as a mouse having entered one of the arms with all four legs.

#### Light Dark Box Test

The apparatus was a rectangular box (50 cm × 30 cm × 30 cm) divided into a smaller (1/3) black area with a lid and a larger (2/3) light area with an open top. A wall separated the two compartments, and a door (5 cm × 5 cm) opened at floor level, which enabled mice to pass. The light intensity was ~500 lx in the light part. Each mouse was placed in the center of the dark compartment (facing away from the door), and behavior was recorded for 5 min. The time spent in the light box and the number of transitions from dark to light compartments were recorded.

#### Y Maze

The apparatus comprised three symmetric and identical arms (34 cm × 8 cm × 15 cm), spaced 120° apart. Each mouse was placed facing the end of one arm and was allowed to explore spontaneously for 10 min. The frequency and order of entries into each arm were analyzed. A correct alternation occurred when the mouse entered the three arms in order, and the alternation index was calculated as: Total correct alternations/(Total arm entries–2) × 100 (%).

#### Tail Suspension Test

Mice were suspended 30 cm above the floor with adhesive tape applied ~2 cm from the end of the tail on a hook. At the beginning of the test, nearly all the mice attempted to escape from hanging, but after a period of struggling, they showed intermittent immobility. The immobility time was recorded in the last 4 min. A longer immobility time served as an indicator of increased depression-like status.

#### Forced Swim Test

The forced swim apparatus was a transparent cylinder (height: 23 cm; diameter: 19 cm) containing 15 cm-high water maintained at ~23°C (BIO-FST-DSM, Bio-seb, FL, USA). Mice were dropped individually into the cylinder and remained there for 5 min. Immobility was judged as the mouse floating in the water, except for small movements to keep its head above the water. The duration of immobility was recorded during the last 4 min. Similarly, a longer immobility time served as an indicator of increased depression-like status.

### Quantification and Statistical Analysis

To do the quantitation analysis, the positive cells were counted, and ratios were calculated by assessors who were blind to the genotype of the mice. Statistical analyses were applied using GraphPad Prism 8 software. All values are expressed as the mean ± SEM. Two-way ANOVA with repeated measures followed by Sidak’s *post hoc* test or two-tailed unpaired Student's *t* test was used to determine statistical significance, as noted in each figure legend. Results were considered significant when the *P* value was < 0.05. The number of samples indicates biological replicates and is indicated in each figure legend.

## Results

### Ncx-*Car3* Neurons are Present in a Distinct Neuronal Population in the Neocortex

**Fig. 1:**
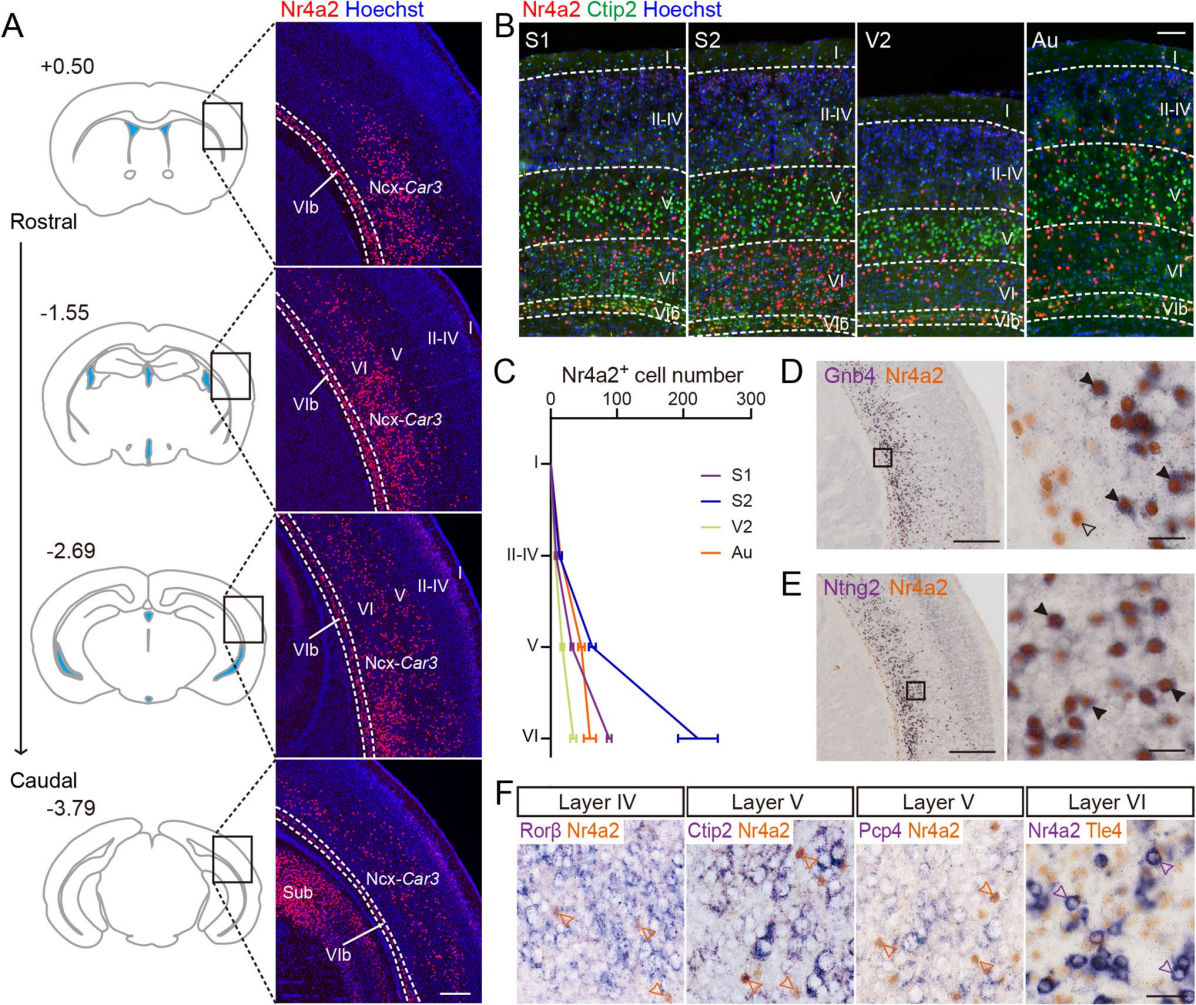
The distribution pattern of Ncx-*Car3* neurons in the lateral neocortex of adult mice. **A** Left: representative coronal sections that contain Ncx-*Car3* neurons along the AP axis. The upper left numbers reflect the distance from Bregma. Right: Images showing the Ncx-*Car3* cells labeled by Nr4a2 immunofluorescence (red). Hoechst (blue) is counterstained. Sub, subiculum; I-VI, cortical layers I-VI. Scale bar, 200 μm. **B, C** Double staining of Nr4a2 (red) and Ctip2 (green) (**B**), and their numbers in different cortical layers at the different levels of lateral neocortex (*n* = 3) (**C**). Hoechst staining (blue). Au, Auditory cortex; I-VI, cortical layers I-VI; S1, primary somatosensory cortex; S2, secondary somatosensory cortex; V2, secondary visual cortex. Scale bar, 100 μm in (**B**). **D, E** Colocalization of *Gnb4* (**D**) and *Ntng2* (**E**) with *Nr4a2* in the neocortex. *Gnb4* (**D**) and *Ntng2* (**E**) (purple in the cytoplasm) were revealed by *in situ* hybridization, and Nr4a2 (brown in the nucleus) is stained with anti-Nr4a2 antibody. An empty arrowhead shows an Nr4a2-single-labeled neuron in layer VIb, and solid arrowheads indicate the double-labeled Ncx-*Car3* cells. Scale bars, 200 μm (left panel) and 10 μm (right panel). **F** Colocalization of *Nr4a2* with *Rorβ*, *Ctip2*, *Pcp4,* and *Tle4* in the neocortex. *Rorβ*, *Ctip2,* and *Pcp4* (purple) are visualized through *in situ* hybridization, and Nr4a2 (brown) is stained with anti-Nr4a2 antibody. For co-labeling of *Nr4a2* and *Tle4*, they are visualized in an opposite way: *Nr4a2* is visualized by *in situ* hybridization (purple), and Tle4 (brown) is stained using anti-Tle4 antibody. The arrowheads indicate Nr4a2-single-labeled Ncx-*Car3* neurons. Scale bar, 20 μm.

We first used immunofluorescence staining to provide a detailed distribution profile of Nr4a2 in the adult brain. Along the anterior-posterior (AP) axis, the vast majority of Nr4a2-positive neurons in the neocortex were located between the levels of Bregma (in mm) +0.50 and –3.79, and more abundantly distributed at the levels ranging from Bregma –1.55 to –2.69, where the somatosensory, visual and auditory cortices are present (Fig. 1A). To further investigate their distribution characteristic in cortical layers, double immunostaining of Nr4a2 and Ctip2 together with Hoechst counterstaining was applied; *Ctip2* (also known as *Bcl11b*) mainly serves as a marker for cortical layer V [18, 19]. As shown in Fig. 1B and C, Ncx-*Car3* neurons were primarily located in layer VI, to a lesser degree in layer V, and even fewer in layers II-IV.

It should be noted that there were two types of Nr4a2^+^ neurons: one with intense signals located dominantly in layers V-VI along the AP axis as described above, and the other with weak signals located in layer II at the caudal one-fourth of the AP extent (from Bregma –2.69 to –3.79) (Fig. 1A, B). In addition, cortical layer VIb also contained Nr4a2^+^ neurons (Fig. 1A, B) as reported in our previous study [15], but they are not included in the *Car3* population. As mentioned, the three groups of *Car3* neurons have similar molecular identity [12, 13, 20, 21], and guanine nucleotide binding protein beta 4 (*Gnb4*) and netrin G2 (*Ntng2*) are known to be specific markers for the CLA and dEn [22, 23]. Our results from a combination of *in situ* hybridization of *Gnb4* or *Ntng2* with immunostaining of Nr4a2 showed that most *Nr4a2*-expressing neurons with intensive immunoreactivity in layers V-VI also expressed these two genes, and *vice versa* (Figs. 1D, E, and S1A, B), and they fit with the classification of *Car3* neurons [12, 13, 20, 22]. On the other hand, those with weak signals in layer II were not labeled with *Gnb4* or *Ntng2,* and thus were not considered to be the *Car3* neurons in this study. To examine if the Ncx-*Car3* ensemble belongs to known populations of cortical neurons, co-labeling of *Nr4a2* with *Rorβ* (a marker for layer IV), *Ctip2* (layer V), *Pcp4* (layer V) and *Tle4* (layer VI) were applied [19, 24], but none of Ncx-*Car3* neurons was co-localized with these genes (Fig. 1F). Taken together, our results indicate that Ncx-*Car3* neurons are a unique neuronal population in the deep layers of the lateral neocortex with a limited AP extent.

### Later-Born Ncx-*Car3* Neurons Have Bipolar Morphology but Settle Down in Deep Layers of the Neocortex

**Fig. 2:**
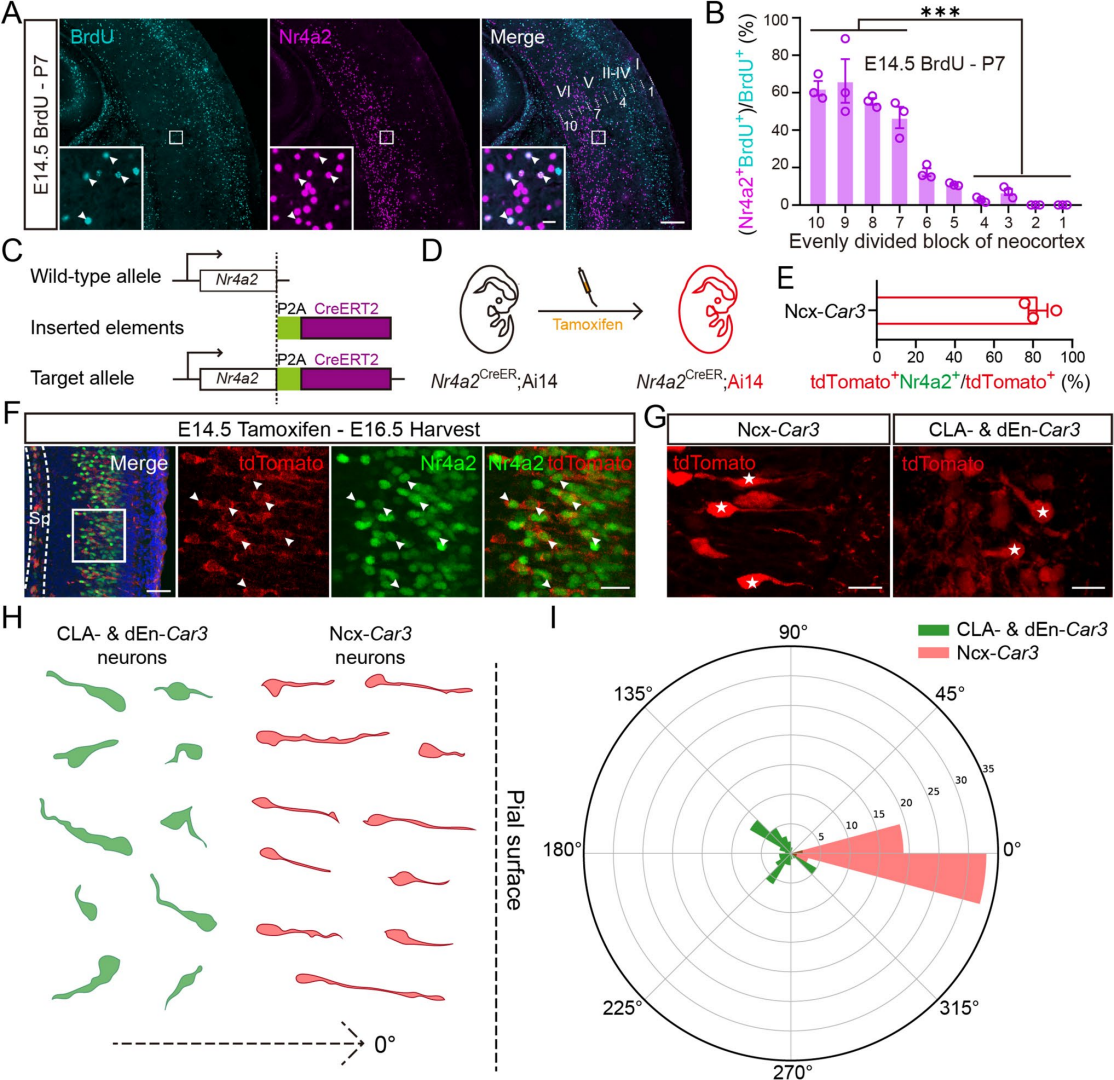
The later-born Ncx-*Car3* cells prefer to settle in deep layers of the neocortex and have bipolar morphology toward the pial surface. **A** Colocalization of BrdU (cyan) and Nr4a2 (magenta) in the neocortex of P7 mice with a single pulse of BrdU injection in pregnant mice at E14.5. The arrowheads indicate double-labeled cells. The neocortex is divided into ten bins from deep to superficial layers (as 10 to 1 in the right panel). I-VI, cortical layers I-VI. Scale bars, 200 μm and 10 μm (inserts). **B** The ratios of (Nr4a2^+^/BrdU^+^ cell number)/(BrdU^+^ cell number) in evenly divided neocortex blocks (as shown in **A**) of P7 mice with BrdU injection at E14.5. *n* = 3. ***P < 0.001. **C** Schematic of establishing the *Nr4a2*^CreER^ mouse line. CreERT2 is transcribed together with *Nr4a2* and then translated into protein separately. **D** Diagram of tamoxifen administration in the embryonic *Nr4a2*^CreER^; Ai14 mouse line. **E, F** Colocalization of Nr4a2 (green) and tdTomato (red) in the neocortex of an E16.5 *Nr4a2*^CreER^; Ai14 mouse (**F**); one pulse of tamoxifen administered at E14.5. The ratio of (tdTomato^+^Nr4a2^+^ cell number)/(tdTomato^+^ cell number) (*n* = 3) (**E**). Arrowheads indicate double-labeled cells in (**F**). Scale bars, 50 μm (left panel) and 20 μm (right three panels) in (**F**). **G** Representative image of presumptive Ncx-, CLA-, and dEn-*Car3* neurons (asterisked) delineated by tdTomato fluorescence. Scale bar, 20 μm. **H** Representative diagram of presumptive Ncx-*Car3* (11 neurons, *n* = 3), and CLA- and dEn-*Car3* neurons (10 neurons, *n* = 3) delineated by tdTomato fluorescence. The direction perpendicular to the pial surface is defined as zero degrees. **I** The directions of presumptive Ncx-*Car3* (55 neurons, *n* = 3), and CLA- and dEn-*Car3* neurons (50 neurons, *n* = 3) are aligned in the same radar graph.

Given the similar transcriptomic identity among the three groups of *Car3* neurons, they might share some common aspects of the developmental process. We first examined their birth date by pulse BrdU labeling at embryonic stages, and the pups were collected at P7. We quantified the proportions of BrdU^+^/Nr4a2^+^ cells in the total of Nr4a2^+^ cells in the neocortex, CLA, and dEn. This showed that the vast majority of Ncx-*Car3* cells were born during E10.5-E14.5 with a peak generation at E12.5, which was different from CLA-*Car3* and dEn-*Car3* neurons that were born during E10.5–12.5 without a peak time point (Fig. S1C, D). Early-born cortical neurons settled down in the deep layer, while later-born neurons migrated radially, passed through the early-born neurons, and occupied the superficial layer. As expected, the vast majority of E14.5 BrdU-labeled later-born neurons were located in superficial layers at P7 (Figs 2A and S1E). However, there were still a small number of BrdU^+^ cells in the deep layers (Fig. S1E). It is worth noting that ~50% of the BrdU^+^ cells remaining in the deep layers expressed *Nr4a2,* while this proportion was <10% in the superficial layers (Fig. 2B). Thus, the later-born Ncx-*Car3* neurons do not strictly follow the inside-out migration to be located in the superficial layers as other cortical neurons generated at the same time.

To further explore the migration of the *Car3* neurons, we generated the *Nr4a2*^CreER^ mouse line and crossed it with the Ai14 reporter mouse line (Fig. 2C, D). One dose of tamoxifen (100 mg/kg) was administered at E14.5, and the embryos were collected two days later. Most Nr4a2^+^ neurons were well visualized with fluorescence (Fig. 2E, F). Notably, the presumptive Ncx-*Car3* neurons showed a bipolar shape and their leading processes exhibited strong angular clustering perpendicular to the pial surface, whereas those located in the CLA and dEn had shorter processes without a consistent or stationary direction (Fig. 2G–I). These results suggest that Ncx-*Car3* neurons seem to be equipped with radial migration morphology but do not keep the same pace as other cortical neurons in migration.

### *Nr4a2* Is Required for the Gene Expression of Ncx-*Car3* Neurons

**Fig. 3:**
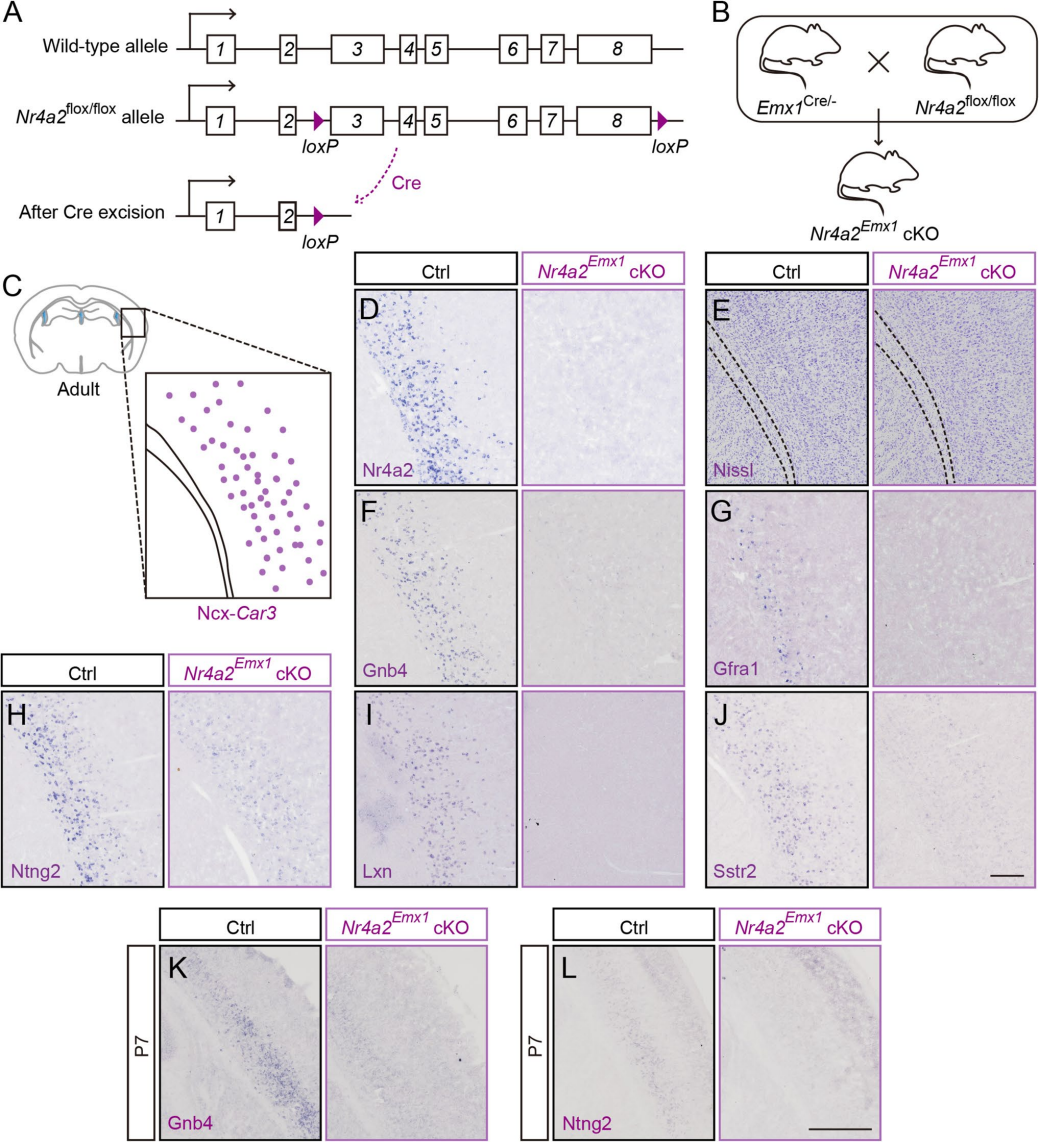
Defective expression of Ncx-*Car3* enriched genes in adult *Nr4a2*^*Emx1*^ cKO mice. **A, B** Schematic of the generation of the *Nr4a2*^flox/flox^ allele construct (**A**) and the deletion of the *Nr4a2* gene with *Emx1*-driven Cre (**B**). **C** Schematic of the representative Ncx-*Car3* region examined, which contains abundant Ncx-*Car3* neurons. **D** *In situ* hybridization showing the deletion of *Nr4a2* in the Ncx-*Car3* region of adult *Nr4a2*^*Emx1*^ cKO mice compared with controls. **E** Nissl staining shows no difference in cortical architecture between adult *Nr4a2*^*Emx1*^ cKO and control mice. **F-J** *In situ* hybridization showing a significant reduction of the expression of *Gnb4* (**F**)*, Gfra1* (**G**), *Ntng2* (**H**), *Lxn* (**I**), and *Sstr2* (**J**) in the Ncx-*Car3* region of adult *Nr4a2*^*Emx1*^ cKO mice compared with control mice. Scale bar, 200 μm (**D–J**). **K, L** *In situ* hybridization showing significant reductions of *Gnb4* (**K**) and *Ntng2* (**L**) expression in the Ncx-*Car3* region of *Nr4a2*^*Emx1*^ cKO mice compared to controls at P7. Scale bar, 500 μm.

Conventional *Nr4a2* knockout mice die within two days after birth [1], while the mice with *Nr4a2* deletion restricted to the forebrain can survive to adulthood. The initiation of *Nr4a2* expression in the presumptive Ncx-*Car3* region is at ~E15.5 (Fig. S2A), which is later than that of the Cre recombinase activity of *Emx1*^Cre^ mice [16]. Thus, *Emx1*^Cre^; *Nr4a2*^flox/flox^ (*Nr4a2*^*Emx1*^ cKO) mice were generated to explore the roles of *Nr4a2* in the development of Ncx-*Car3* neuron (Fig. 3A–D). Nissl staining showed that the layered architecture of the neocortex was not changed in adult cKO mice (Fig. 3E). Moreover, the expressions of several layer-specific genes, including *Lmo4*, *Ctip2*, *Lhx2*, *PlxnD1*, *Pcp4,* and *Foxp2,* were not altered compared with littermate controls (Fig. S3). On the other hand, *Gnb4*, *Gfra1*, *Ntng2*, *Lxn,* and *Sstr2,* which are uniquely expressed by the Ncx-*Car3* neurons in the neocortex, were undetectable in the *Nr4a2*^*Emx1*^ cKO mice (Fig. 3F–J). Among these altered genes, the expression of *Gfra1*, *Lxn,* and *Sstr2* in Ncx-*Car3* neurons was late, and their decrease was not evident until one month in *Nr4a2*^*Emx1*^ cKO mice (data not shown), whereas the decrease of Gnb4 and Ntng2 expression was detectable in *Nr4a2*^*Emx1*^ cKO mice as early as P7 (Fig. 3K, L). Taken together, *Nr4a2* is required for the normal gene expression of Ncx-*Car3* neurons during postnatal development.

**Fig. 4:**
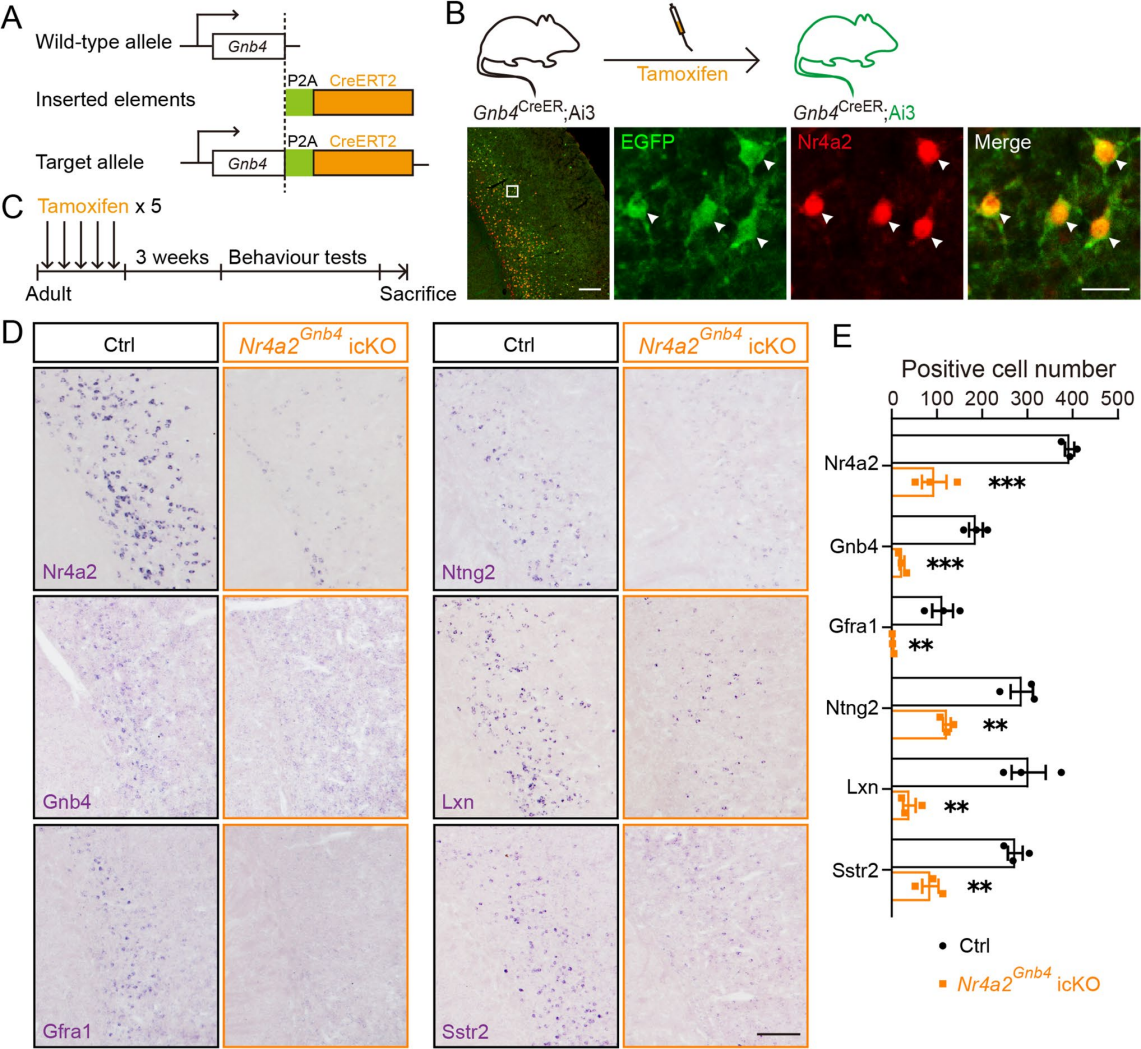
The number of cells expressing Ncx-*Car3* enriched genes is significantly decreased in *Nr4a2*^*Gnb4*^ icKO mice. **A** Schematic of establishing the *Gnb4*^CreER^ mouse line. CreERT2 is transcribed together with *Gnb4* and then translated into protein separately. **B** Diagram of tamoxifen administration in adult *Gnb4*^CreER^; Ai3 mouse line (upper), and the effectiveness of Cre activity is demonstrated by colocalization of EGFP (green) and Nr4a2 (red). Arrowheads indicate double-labeled cells. Scale bars, 200 μm (left panel) and 20 μm (right three panels). **C** Diagram of tamoxifen administration in adult *Nr4a2*^*Gnb4*^ icKO mice. **D, E** *In situ* hybridization showing significant reductions in the numbers of cells expressing *Nr4a2*, *Gnb4, Gfra1*, *Ntng2*, *Lxn,* and *Sstr2* in the Ncx-*Car3* region of adult *Nr4a2*^*Gnb4*^ icKO mice compared with age-matched controls. *n* = 3. **P < 0.01, ***P < 0.001. Scale bar, 200 μm in (**D**).

*Nr4a2* expression in Ncx-*Car3* neurons is persistent in adulthood. It is of interest to investigate if *Nr4a2* is also involved in the maintenance of gene expression in mature brains. As mentioned above, *Gnb4* is selectively expressed by *Car3* neurons in the forebrain (Fig. 1D), and the *Gnb4*^CreER^ mouse line was generated to delete *Nr4a2* in the Ncx-*Car3* neurons in a time-controlled manner (Fig. 4A). Similarly, after testing the efficiency and specificity of Cre activity through the *Gnb4*^CreER^; Ai3 reporter mouse line (Fig. 4B), two-month-old *Gnb4*^CreER^; *Nr4a2*^flox/flox^ (*Nr4a2*^*Gnb4*^ icKO) mice were given 5 doses of tamoxifen (200 mg/kg i.g.) in a two-day interval (Fig. 4C). Deletion of *Nr4a2* was confirmed, as shown by ~76% reduction of Nr4a2^+^ neurons in *Nr4a2*^*Gnb4*^ icKO compared with control mice (Fig. 4D, E). Interestingly, *Gnb4*-, *Gfra1*-, *Ntng2*-, *Lxn*-, and *Sstr2*-expressing neurons were significantly decreased in number in the region containing Ncx-*Car3* neurons of *Nr4a2*^*Gnb4*^ icKO mice compared to control mice (Fig. 4D, E).

To determine whether the reduced expression level of genes was caused by the loss of neurons, we immunostained for apoptosis-related marker cleaved-caspase3 in P0- and P3-*Nr4a2*^*Emx1*^ cKO mice, and in adult *Nr4a2*^*Gnb4*^ icKO mice (2 weeks and 3 months after tamoxifen administration). There was no significant difference in the number of cleaved-caspase3-positive cells in cKO and icKO mice compared with their respective controls (Fig. S4A–C, E). Furthermore, Nissl staining at the above time points also reflected no significant differences between cKO/icKO and their littermate controls (Fig. S4A, B, D, F). Together with the data from *Nr4a2*^*Emx1*^ cKO mice, we concluded that Nr4a2 serves as a key regulator of the transcriptomic profile of the Ncx-*Car3* population during development and after maturity.

### *Nr4a2* Is Sufficient to Control the Gene Expression of Ncx-*Car3* Neurons *in Vivo*

**Fig. 5:**
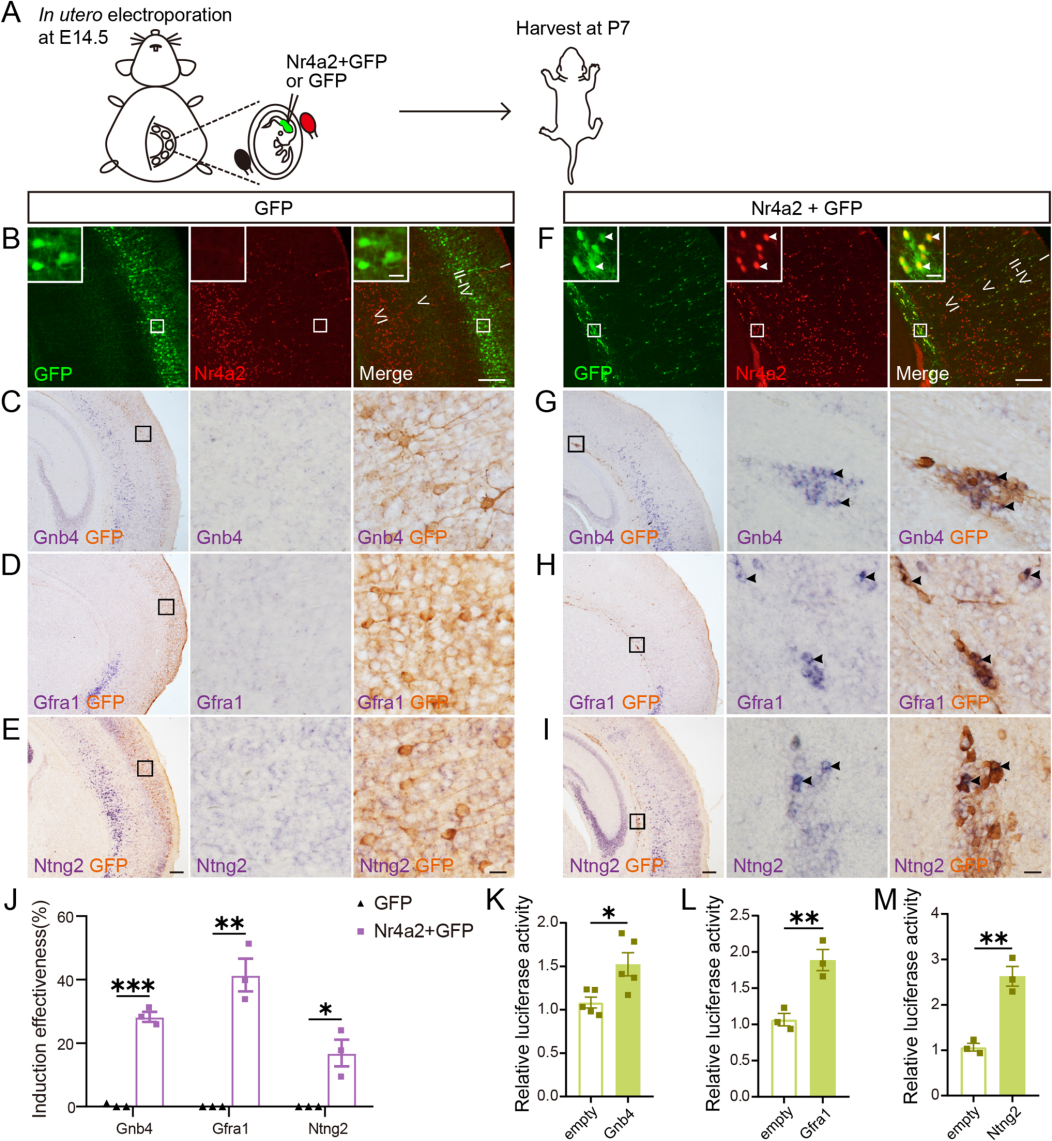
*Nr4a2* is sufficient to induce the expression of Ncx-*Car3-*enriched genes *in vivo.* **A** Diagram of *Nr4a2* misexpression in wild-type mice by *in utero* electroporation at E14.5 and examined at P7. **B–E** Expression of GFP does not induce expression of *Gnb4*, *Gfra1,* and *Ntng2* in the neocortex of wild-type mice. GFP-expressing neurons (green in **B**, brown in **C–E**) are primarily located in the superficial layers, and they do not contain Nr4a2 (red in **B**) or mRNA for *Gnb4* (**C**), *Gfra1* (**D**), and *Ntng2* (**E**)*.* Note that endogenous expression of these genes is seen in the neocortex. I-VI, cortical layers I-VI. Scale bars, 200 μm (**B** and left panel in **C–E**) and 10 μm (inserts in **B** and right two panels in **C–E**). **F-I** Misexpression of *Nr4a2* does induce ectopic expression of *Gnb4*, *Gfra1,* and *Ntng2* in the neocortex of wild-type mice. Misexpression of Nr4a2 (red) is confirmed by immunostaining (arrowheads in inserts, **F**), and these neurons are located in the deepest cortical regions instead of the superficial layers (**F**). Ectopic expression of *Gnb4* (**G**), *Gfra1* (**H**), and *Ntng2* (**I**) (purple) detected by *in situ* hybridization is evident in *Nr4a2*-misexpressing neurons labeled by GFP (brown). To present mRNA signals, *in situ* hybridization for individual genes was done first and photographed (middle panels, **G–I**). Arrowheads indicate the double-labeled neurons. Scale bars, 200 μm (**F** and left panel in **G–I**) and 10 μm (inserts in **F** and right two panels in **G–I**). **J** The induction effectiveness is shown by calculating the ratio of double-labeled cell number/GFP^+^ cell number. *n* = 3. *P < 0.05, **P < 0.01, ***P < 0.001. **K–M** Dual-luciferase reporter assays showing the luciferase activity of *Gnb4* (**K**), *Gfra1* (**L**), and *Ntng2* (**M**) is significantly increased in the presence of *Nr4a2*. *n* = 5 per group in (**K**) and *n* = 3 per group in (**L**, **M**) for statistics. *P < 0.05, **P < 0.01.

We next examined whether *Nr4a2* is sufficient in the regulation of Ncx-*Car3* neuron gene expression. To this end, *Nr4a2* was misexpressed in the cerebral cortex of wild-type mice *via in utero* electroporation at E14.5, and pups were examined at P7 (Fig. 5A). Misexpressing *Nr4a2* affected cortical neuron migration as shown by the presence of these neurons in the deepest regions of the neocortex in comparison to those expressing GFP only (Fig. 5B–I). Critically, *Gnb4*, *Gfra1,* and *Ntng2* were ectopically induced in these neurons (Fig. 5G–J). In addition, Nr4a2 acts as a transcription factor and directly regulates the expression of target genes [25, 26]. To examine whether the Nr4a2 protein can bind to the promoter regions of those genes expressed in Ncx-*Car3* neurons, we applied the dual-luciferase reporter assay and found that *Nr4a2* overexpression had a significant increase in luciferase activity (Fig. 5K–M). These results illustrate that Nr4a2 directly binds to the promoter regions of Ncx-*Car3*-enriched genes (i.e., *Gnb4*, *Gfra1,* and *Ntng2*), through which it regulates their expression.

### Hyperactivity and Reduced Anxiety-like Behaviors Are Present in Both ***Nr4a2***^***Emx1***^ cKO and ***Nr4a2***^***Gnb4***^ icKO Mice

**Fig. 6:**
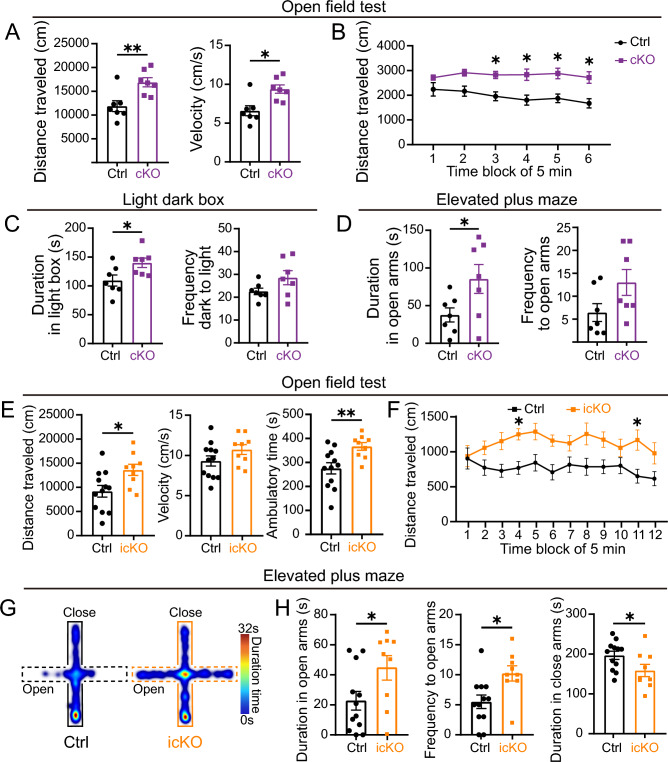
Both *Nr4a2*^*Emx1*^ cKO and *Nr4a2*^*Gnb4*^ icKO mice present hyperactivity and low anxiety-like behaviors. **A, B** In the open field test, *Nr4a2*^*Emx1*^ cKO mice display increased distance traveled and velocity compared with controls (**A**). Note that the cKO mice exhibit a stationary increase in locomotion while control mice show a gradually decreasing trend during the 30-min testing period (**B**). *n* = 7. *P < 0.05, **P < 0.01. **C** In the light-dark box test, the duration in the light box of *Nr4a2*^*Emx1*^ cKO mice is significantly longer than that of controls (left panel), and the transition frequency from dark to light box trends to increase, although it does not reach statistical significance (right panel). *n* = 7. *P < 0.05. **D** In the elevated plus maze test, the duration in the open arms of *Nr4a2*^*Emx1*^ cKO mice is significantly longer than that of controls (left panel), and the transition frequency to open arms also has a tendency to increase, although it does not reach statistical significance (right panel). *n* = 7. *P < 0.05. **E, F** In the open field test, *Nr4a2*^*Gnb4*^ icKO mice present a significantly increased distance traveled and ambulatory time compared with controls (first and third panels in **E**). The velocity of icKO mice tends to rise, although it does not reach a statistically significant difference (middle panel in **E**). Similarly, the icKO mice show a stationary increase in locomotion while control mice show a trend to gradually decrease during the 60-min testing period (**F**). *n* = 9. *P < 0.05, **P < 0.01. **G, H** In the elevated plus maze test, *Nr4a2*^*Gnb4*^ icKO mice spend more time in and enter more times into the open arms. Correspondingly, the duration in the closed arms of icKO mice is decreased (**H**). Representative heatmaps of *Nr4a2*^*Gnb4*^ icKO and control mice in the elevated plus maze test are shown in (**G**). *n* = 9. *P < 0.05, **P < 0.01.

Having found that the deletion of *Nr4a2* leads to defective gene expression in the Ncx-*Car3* neurons during their development and also in adulthood, implying that the loss of function may potentially affect brain functions. In the open field test, *Nr4a2*^*Emx1*^ cKO mice traveled a much longer distance with increased velocity compared with control mice in the 30-min testing period (Fig. 6A, B). In addition, the *Nr4a2*^*Emx1*^cKO mice spent more time in the light compartment in the light-dark box test and the open arms in the elevated plus maze test, and increased frequency of entry into the light box and the open arms, although it did not reach a statistically significant difference (Fig. 6C, D), suggesting less anxiety-like behavior than control mice. However, the performance in the Y-maze, tail suspension, and forced swim tests showed no differences compared with controls (Fig. S5A–C). These data support the conclusion that *Nr4a2* is involved in regulating some aspects of mouse behavior.

Since compensatory events may occur after the gene manipulation during embryonic development, and therefore, unknown defects may be present in *Nr4a2*^*Emx1*^cKO mice, this may lead to misinterpretation of Nr4a2’s role in regulating brain functions. In addition, *Nr4a2* is also expressed in layer VIb and the subiculum, where Emx1-driven Cre is also present. To avoid these limitations, we then examined the performance of *Nr4a2*^*Gnb4*^ icKO mice after 5-dose tamoxifen induction in adulthood (Fig. 4C). To further confirm the phenotype and reveal the long-lasting hyperactive state after *Nr4a2* deletion, we applied an extended open field test, which lasted 1 h. As shown in Fig. 6E and F, an increased distance traveled and ambulatory time were also found in *Nr4a2*^*Gnb4*^ icKO mice, with a trend of increased velocity compared with control mice. Next, we carried out the light-dark box and elevated plus maze tests. Although there was no difference between the two groups in the light-dark box test (Fig. S5D), the frequency of entry into and time spent in the open arms were increased in *Nr4a2*^*Gnb4*^ icKO mice in the elevated plus maze test (Fig. 6G, H). Based on these results, we propose that the Ncx-*Car3* population is very likely to be implicated in the modulation of anxiety-like behaviors and locomotor activity in mice.

## Discussion

Ncx-*Car3*, as a special subclass of cortical excitatory neurons, has been revealed by several scRNA sequencing assays in recent years [12, 13, 20, 21] and has attracted great attention so far. Peng *et al.* fully reconstructed single Ncx-*Car3* neurons and found that all these neurons project extensively within the cerebral cortex [13]. However, little is known about the underlying mechanism of the developmental process. *Nr4a2*-expressing neurons in the neocortex are thought to compose the *Car3* population according to the recent scRNA sequencing data [27], while there was no morphological evidence to confirm this. Here, we showed a detailed characterization of Nr4a2^*+*^ neurons in the lateral neocortex.

These Ncx-*Car3* neurons are primarily situated in layers V-VI of the lateral neocortex, at the levels from Bregma +0.50 to –3.79, and are positioned at a considerable distance from the CLA and dEn (Fig. 1A–C). Through immunostaining, we found that these neurons do not belong to the major known neuronal populations (i.e., *Rorβ*, *Ctip2*, *Pcp4,* and *Tle4*). It is noteworthy that most E14.5 BrdU-labeled Ncx-*Car3* neurons settle in deep layers instead of superficial layers. In addition, Ncx-*Car3* neurons display similarity with both the CLA- and dEn-*Car3* neurons at the transcription level, as shown by the expression of *Gnb4* and *Ntng2,* the unique markers for CLA- and dEn-*Car3* neurons [22, 23]. The dEn-*Car3* and CLA-*Car3* neurons are generated mostly between E10.5–12.5, and our data showed that the Ncx-*Car3* neurons are born at E11.5–13.5 predominantly, which is consistent with the gradient neurogenesis phenomenon reported in rodents [28]. Taken together, our results fully support the recent scRNA assay data that Ncx-*Car3* neurons compose a distinct neuronal population in the cerebral cortex [13].

So far, the origin of Ncx-*Car3* neurons has not been addressed. There is speculation that Ncx-*Car3* neurons migrate dorsally from the CLA anlage to reach the dorsal pallium [29] and initiate expression of *Lxn* and *Nr4a2* [30]. The distinct molecular characteristics of Ncx-*Car3* neurons from neighboring cortical neurons seem to add to some mysteries of their origin. To delineate the migration process of Ncx-*Car3* neurons, we established the *Nr4a2*^CreER^; Ai14 reporter mouse line and attempted to trace Ncx-*Car3* neurons at the embryonic stage (Fig. 2C, D). We administered one dose of tamoxifen at E14.5 and harvested the pups at E16.5. Remarkably, almost all Ncx-*Car3* cells had a long leading process toward the pial surface (Fig. 2G). In contrast, the presumptive CLA-*Car3* and dEn-*Car3* cells showed more and shorter processes without uniform orientation (Fig. S1C). Notably, we did not observe any tangentially migrating Ncx-*Car3* cells in the entire neocortex. A possible explanation for this is that the Ncx-*Car3* neurons may be generated in the same ventricular zone in the lateral pallium as CLA-*Car3* and dEn-*Car3* neurons, but they migrate radially instead of ventrally [31, 32]. It would be interesting to address the migration process of Ncx-*Car3* neurons.

In this study, we present evidence that *Nr4a2* is required and sufficient for the normal expression of *Car3*-enriched genes during embryonic development and in adulthood. However, the ectopic induction of *Car3*-enriched genes in the cortex of wild-type mice was only achieved at the embryonic stage (Fig. 5) but not in P0 or adult brain (data not shown). The different induction capabilities of *Nr4a2* may be explained by a large gap in the plasticity between newborn and maturing or mature neurons. However, overexpression of *Nr4a2* in the anterior cingulate cortex can reverse depressive- and anxiety-like behaviors caused by LPS administration [10], and overexpressed *Nr4a2* in the primary motor cortex helps to alleviate the motor dysfunction induced by intracerebral haemorrhage in the striatum [33]. Although our data demonstrated that the misexpression of *Nr4a2* in the postnatal brain is unable to induce ectopic expression of *Car3*-enriched genes, its overexpression in adult neurons may alter the expression of some genes that are associated with the functions of Nr4a2^+^ neurons, making it potentially a therapeutic approach for related diseases beyond the midbrain dopaminergic system.

Ncx-*Car3* neurons are reported to have extensively projecting targets in the brain [13], implying their large-scale neuromodulatory roles in brain functions. In this study, we applied a series of behavioral tests for both *Nr4a2*^*Emx1*^ cKO and *Nr4a2*^*Gnb4*^ icKO mice. As expected, *Nr4a2*^*Emx1*^ cKO mice exhibited hyperactivity and reduced levels of anxiety-like behavior. In addition to the *Car3* populations, *Nr4a2* is also expressed in cortical layer VIb, subiculum, hippocampus, and entorhinal cortex, which are also inactivated in *Nr4a2*^*Emx1*^ cKO mice; therefore, these behavioral changes cannot be attributed only to the loss of *Nr4a2* in the *Car3* neurons. We thus applied the behavioral tests in *Nr4a2*^*Gnb4*^ icKO mice to validate the precise role of *Nr4a2* in the forebrain *Car3* populations. Overall, *Nr4a2*^*Gnb4*^ icKO mice had similar alterations in the open field and elevated plus maze tests, indicating their impaired locomotor control and low anxiety-like states in the absence of *Nr4a2*. Among the regions containing *Car3* populations, the CLA is reported to be involved in stress-induced anxiety responses and adolescent cocaine exposure-induced anxiety-like behaviors [34–36], while the dEn has proved to be associated with novelty exploration [37]. Considering the different behavioral changes between our *Nr4a2*^*Gnb4*^ icKO mice and those obtained by virus-mediated deletion of *Nr4a2* in the CLA and dEn [27], we tend to propose that Ncx-*Car3* neurons are very likely to be involved in regulating novelty-evoked locomotion and anxiety. It should be noted that the increased travel distance and movement time in the open field test are also thought to relate to the increased exploring capability of animals, as well as the increased duration in open arms in the elevated plus maze test [38–40]. More studies are needed to clearly define the roles of Ncx-*Car3* neurons in the regulation of brain functions in the future.

In conclusion, our study delineates the developmental process of Ncx-*Car3* neurons and addresses the pivotal role of *Nr4a2* in the development and functional maintenance of this unique subpopulation of cortical neurons.

## Supplementary Information

Below is the link to the electronic supplementary material.

Supplementary file1 (PDF 2103 KB)
