# Supplementary material for: Nr4a2, A Key Factor Controlling the Development and Functional Maintenance of Forebrain Car3 Neurons
Source: Neurosci Bull. 2025 Sep 8;42(3):649–62. doi: 10.1007/s12264-025-01496-z (PMC12950122; doi:10.1007/s12264-025-01496-z)
Supplement: Supplementary file 1 — Supplementary file1 (PDF 2103 KB) [file 12264_2025_1496_MOESM1_ESM.pdf]

Supplementary Materials

Supplementary Figures and Figure Legends

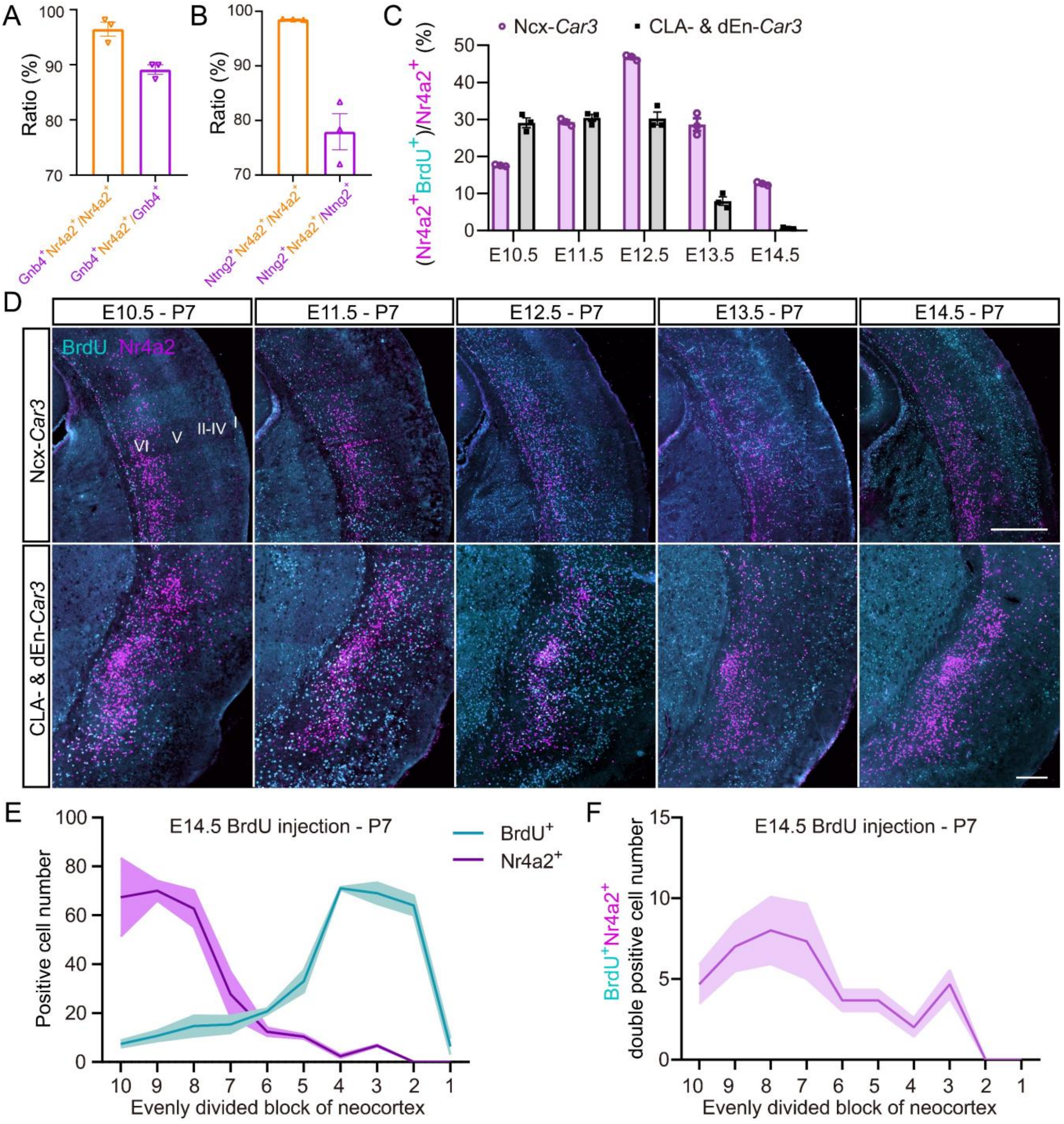

Fig. S1 The co-labeling of Nr4a2 and Gnb4 and Ntng2 in neocortex and the birth date characteristic of *Car3* populations. **A** The co-labeling ratio of (Gnb4$^+$/Nr4a2$^+$ cell number)/ (Nr4a2$^+$ cell number) and (Gnb4$^+$/Nr4a2$^+$ cell number)/ (Gnb4$^+$ cell number) is shown. n = 3. **B** The statistical result of the

co-labeling ratio of (Ntng2$^+$/Nr4a2$^+$ cell number)/ (Nr4a2$^+$ cell number) and (Ntng2$^+$/Nr4a2$^+$ cell number)/ (Ntng2$^+$ cell number). n = 3. **C** The ratios of (Nr4a2$^+$BrdU$^+$cell number)/ (Nr4a2$^+$ cell number) in the Ncx-*Car3* and the other *Car3* regions (CLA- and dEn-*Car3*) are calculated. n = 3. **D** Colocalization of BrdU (cyan) and Nr4a2 (magenta) in Ncx-, CLA- and dEn-*Car3* regions of P7 mice with a single pulse of BrdU injection in pregnant mice at E10.5, E11.5, E12.5, E13.5, and E14.5, respectively. I-VI, I-VI cortical layers. Scale bars, 500 μm (upper panel) and 200 μm (lower panel). **E, F** BrdU-single positive (**E**), Nr4a2-single positive (**E**), and BrdU$^+$/Nr4a2$^+$-double positive cell number (**F**) in P7 neocortex (with BrdU injection at E14.5) are counted and calculated, respectively. The neocortex is evenly divided into ten bins from deep to superficial cortex (shown as 10 to 1 in **Fig. 2A**). The wide bands in light colours around the curves are the error bands that represent SEM. n = 3.

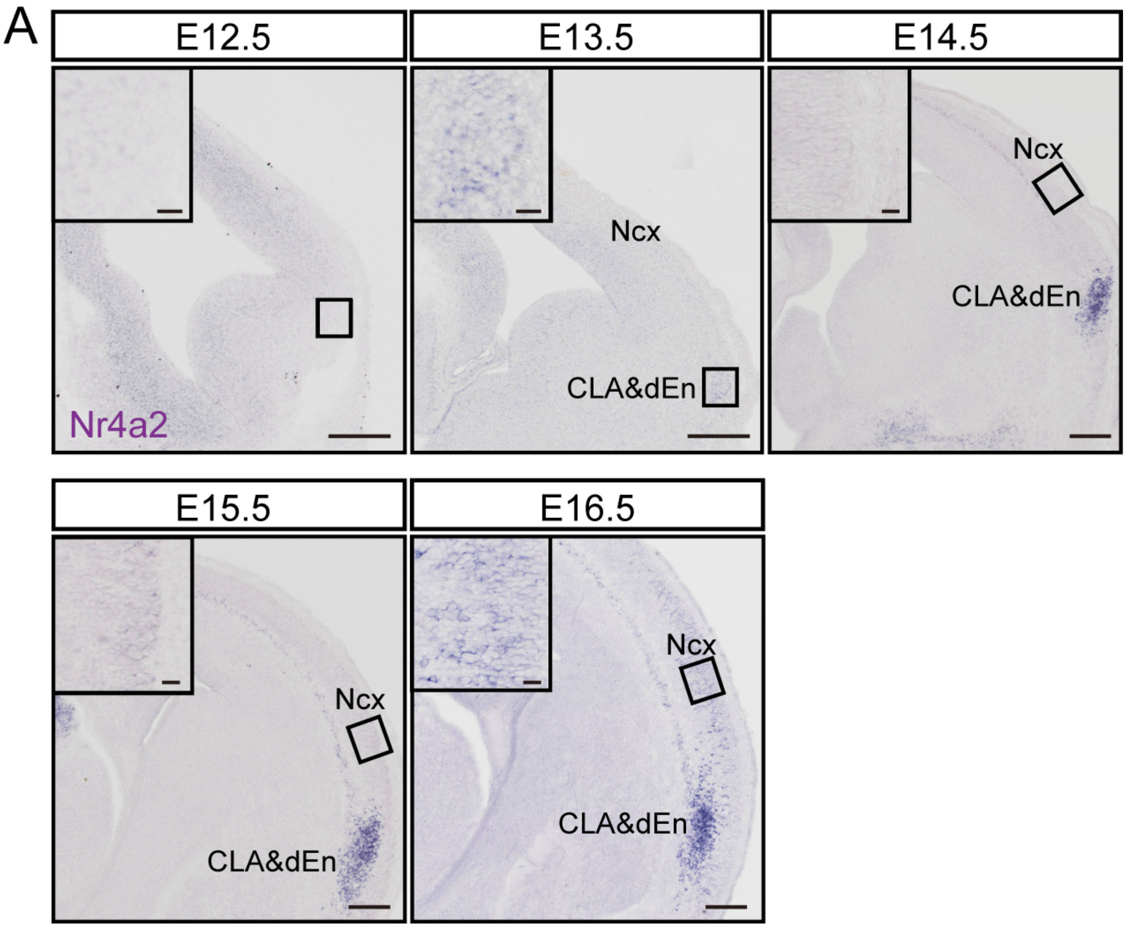

Fig. S2 The expression of *Nr4a2* in the developing cerebral cortex. **A** The expression of *Nr4a2* in the neocortex at different embryonic stages. *Nr4a2* is visualized by *in situ* hybridization. Ncx, neocortex. CLA&dEn, claustrum, and dorsal endopiriform nucleus. Scale bars, 200 µm and 20 µm (inserts).

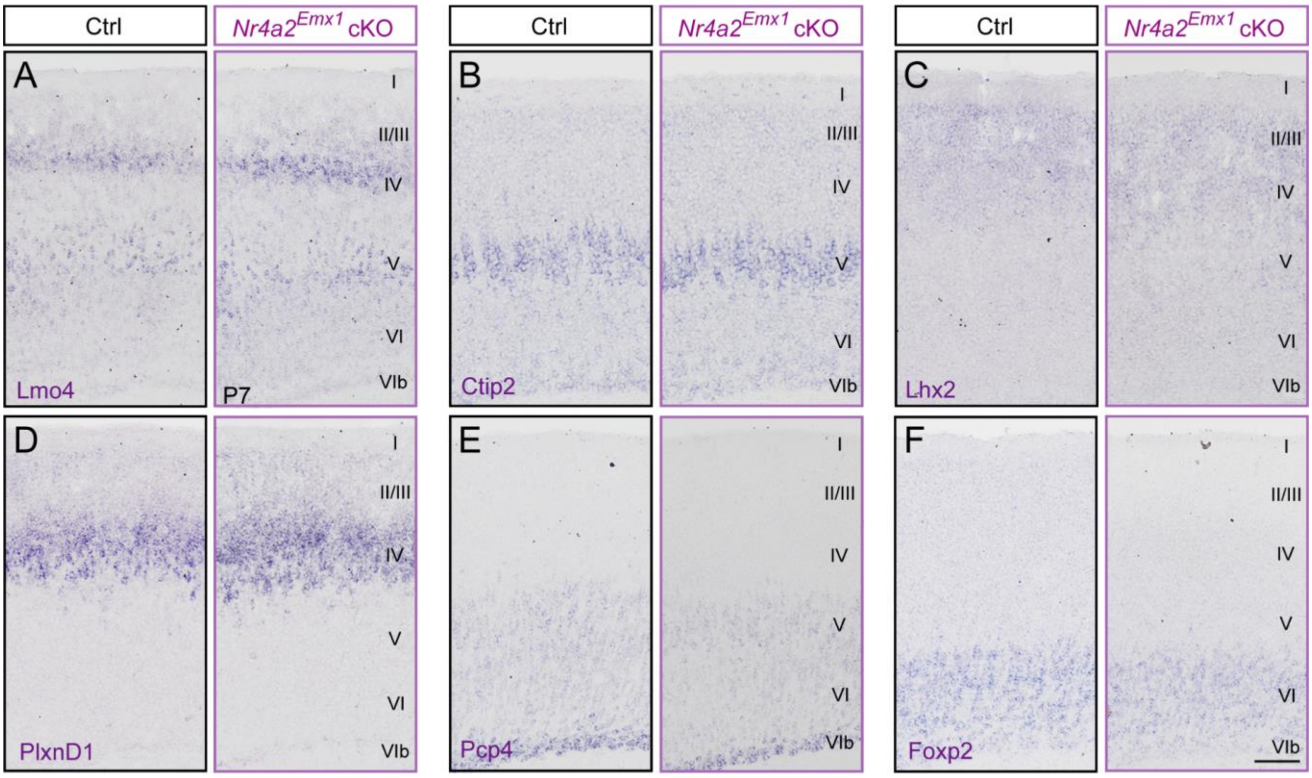

Fig. S3 The expression of layer-specific marker genes in the adult cerebral cortex of *Nr4a2*^{Emx1} cKO and control mice. **A–F** The expression of *Lmo4* (**A**), *Ctip2* (**B**), *Lhx2* (**C**), *PlxnD1* (**D**), *Pcp4* (**E**), and *Foxp2* (**F**) in the neocortex of adult *Nr4a2*^{Emx1} cKO and control mice, which is visualized by *in situ* hybridization. I-VI and VIb, I-VI and VIb cortical layers. Scale bars, 100 µm.

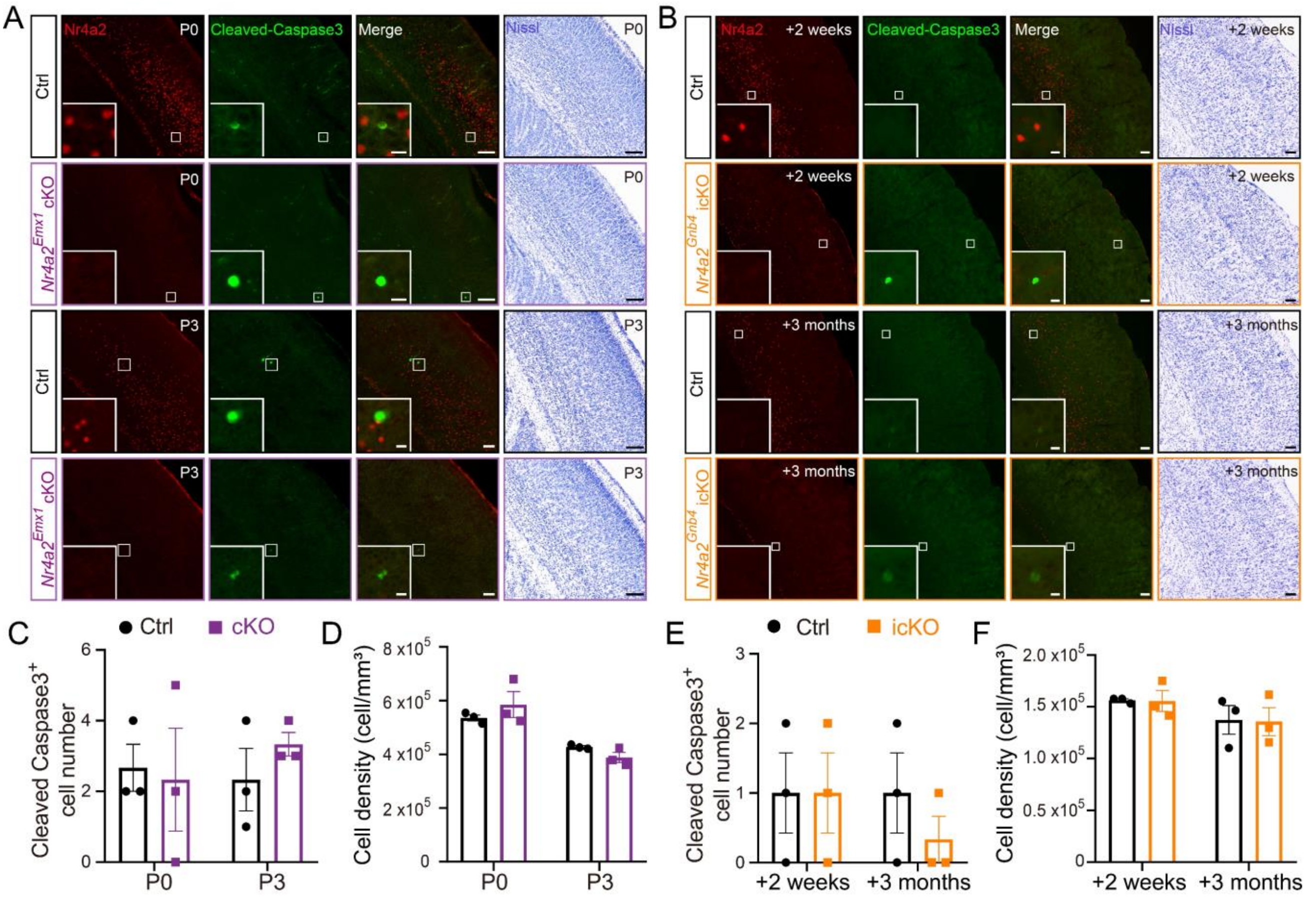

Fig. S4 No abnormal cell apoptosis occurred in the cerebral cortex after Nr4a2 deletion at the embryonic stage and in adulthood. **A** Nissl staining (right panel) and immunostaining of Nr4a2 (red) and cleaved-caspase3 (green) in the neocortex of P0- and P3- *Nr4a2*^{Emx1} cKO and control mice (left three panels). Scale bars, 100 μm and 10 μm (inserts). **B** Nissl staining (right panel) and immunostaining of Nr4a2 (red) and cleaved-caspase3 (green) in the neocortex of adult *Nr4a2*^{Gnb4} icKO and control mice (two weeks and three months after tamoxifen administration; left three panels). Scale bars, 100 μm and 10 μm (inserts). **C, E** Comparisons of the cell numbers of cleaved-caspase3-positive cells in the neocortex of P0- and P3 -*Nr4a2*^{Emx1} cKO and control mice (**C**), and adult *Nr4a2*^{Gnb4} icKO mice (two weeks and three months after tamoxifen administration) with their respective controls (**E**, n = 3). **D, F** Comparisons of the cell density displayed by Nissl staining between P0- and P3 -*Nr4a2*^{Emx1} cKO and control mice (**D**), and between adult *Nr4a2*^{Gnb4} icKO mice (two weeks and three months after

tamoxifen administration) and control mice (**F**, *n* = 3).

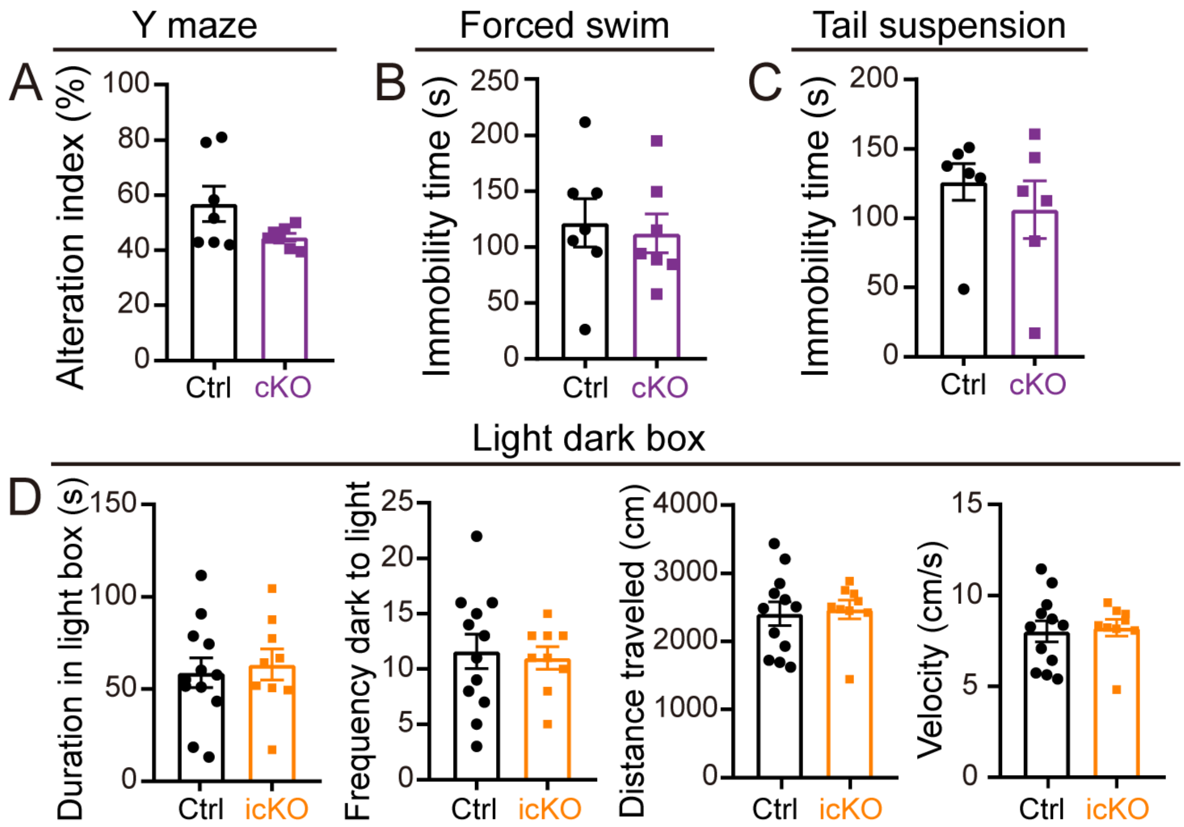

Fig. S5 *Nr4a2*^{Emx1} cKO mice show no difference in working memory and depression-like behaviors, while *Nr4a2*^{Gnb4} icKO mice show no obvious difference in the light-dark box test related to controls. **A–C** The performance of *Nr4a2*^{Emx1} cKO mice in the Y maze (**A**), forced swim (**B**), and tail suspension tests (**C**) shows no obvious difference compared with controls. *n* = 7 (**A**, **B**) and *n* = 6 (**C**). **D** The performance of *Nr4a2*^{Gnb4} icKO mice in the light-dark box test is comparable to that of controls. *n* = 9.
